# Risk factors for postoperative mortality in congenital diaphragmatic hernia: a single-centre observational study

**DOI:** 10.1007/s00383-016-4032-9

**Published:** 2016-12-16

**Authors:** Darya Kadir, Helene Engstrand Lilja

**Affiliations:** 1 Department of Women’s and Children’s Health, Section of Pediatric Surgery, Uppsala University, 751 85 Uppsala, Sweden; 2 Department of Pediatric Surgery, University Children’s Hospital, Uppsala, Sweden

**Keywords:** Congenital diaphragmatic hernia, Postoperative outcome, Risk factors

## Abstract

**Background:**

The management of congenital diaphragmatic hernia (CDH) is a major challenge. The mortality is dependent on associated malformations, the severity of pulmonary hypoplasia, pulmonary hypertension and iatrogenic lung injury associated with aggressive mechanical ventilation. The aims of the study were to investigate the mortality over time in a single paediatric surgical centre, to compare the results with recent reports and to define the risk factors for mortality.

**Methods:**

The medical records of infants with CDH from two time periods: 1995–2005 and 2006–2016 were reviewed. Cox regression was used for statistical analysis.

**Results:**

The study included 113 infants. The mortality rate was significantly decreased in the later time period, compared to the earlier, 4.4 and 17.9%, respectively. At the early time period five patients (7.5%) were treated with ECMO and in the later time period ECMO was used in three patients (6.5%). The mortality in ECMO-treated patients was 50% in both time periods. Prenatal diagnosis, intrathoracic liver, low Apgar score and low birth weight were defined as independent risk factors for mortality.

**Conclusion:**

Despite no significant differences in the incidence of independent risk factors and the use of ECMO between the two time periods, mortality decreased over time. The mortality was lower than previously reported. The results indicate that there are many important factors involved in a successful outcome after CDH repair. Large multicentre studies are necessary to define those critical factors and to determine optimal treatment strategies.

## Introduction

The management of congenital diaphragmatic hernia (CDH) is a major challenge in paediatric surgery. The incidence of CDH is between 1 in 2500 and 5000 live births [1–3]. A mortality rate of 10–30% has been reported but is still higher in patients with severe pulmonary hypoplasia and pulmonary hypertension [4–8]. Mortality in CDH patients treated with extracorporeal membrane oxygenation (ECMO) was approximately 50% in a recent report from ELSO (Extracorporeal Life Support Organisation) [9]. Prenatal diagnosis, LHR (lung-to-head-ratio) *n* < 1, intrathoracic liver, right-sided CDH, associated malformations, a large diaphragmatic defect, low 5-min Apgar score and prematurity are other factors that have been associated with high mortality [2, 3, 8, 10–16]. Also the timing of surgery might influence the mortality rate in high-risk CDH and the practice of clinical stability before surgery is widely advocated [10, 17, 18]. The aims of the study were to investigate the mortality over time in a single paediatric surgical centre, to compare the results with recent reports and to define the risk factors for mortality.

## Patients and methods

This is a retrospective observational study of all the infants with repaired CDH between January 1995 and January 2016 at the University Children’s Hospital, Uppsala, Sweden. Neonates primarily managed in our centre who were sent for ECMO to other paediatric surgical centres were also included. Patients with primary repair in other centres who later had a repair of recurrent CDH in our centre and patients not surviving until surgery were excluded. Patients with diaphragmatic eventration were excluded. The use of patient data in this study was approved by the Regional Committee on Medical Research Ethics (Dnr 2016/140). Data extracted from the medical records were prenatal diagnosis, LHR, liver in thorax, gender, gestational age and birth weight, Apgar score, treatment with ECMO, age at surgery (days), side of the defect, associated malformations, the size of the defect as determined by the surgeon at the time of surgery and if a patch was used. If the patient died, the age at death was registered. The study period was 900 days after surgery or until death. A major cardiac anomaly was defined according to Spitz (congenital heart disease that required medical or surgical treatment) [19].

## Statistical analysis

Descriptive statistics are presented as median and range for continuous variables and as absolute and relative frequencies for categorical variables. Survival is presented as Kaplan–Meier curves. All patients that underwent CDH repair were included in the Kaplan–Meier estimate, regardless of the length of follow-up time. Possible independent risk factors for mortality were analysed in three steps. In the first step, selected independent factors had been evaluated using univariate Cox proportional hazards model presented as Hazard ratio (HR) with 95% confidence interval (CI). In the second step, variables with a *p* value <0.05 in the univariate analysis were entered into a multivariate analysis. In the third step, the model was subject to a backward stepwise procedure where the best model was determined as the model with the smallest Akaike information criteria (AIC) value. Estimates where *p* < 0.05 were considered significant, but since no adjustment for multiplicity has been performed the overall type I error rate may exceed 5%. Thus, the *p* values should be interpreted in an exploratory manner rather than as confirmatory. Statistical analyses were performed using R version 3.2.2, Copyright (C) 2015 The R Foundation for Statistical Computing.

## Results

A total of 113 patients were included, 67 in the early time period (1995–2005) and 46 in the later time period (2006–2016) (Table 1). The distribution of males and females was similar in both time periods. Median gestational age was 38 weeks in both time periods and birth weight was 3192 g in the early and 3215 g in the later time period. Median age at surgery was 2 days in the early and 3 days in the later time period. The incidence of major cardiac anomaly was 13.4% in the early and 26.1% in the later time period and the incidence of chromosomal abnormalities was 3% in the early and 2.2% in the later time period. The frequencies of other malformations such as esophageal atresia, gastroschisis, bladder exstrophy, omphalocele, myelomeningocele, Pentalogy of Cantrell, Fryns- and Pierre Robin syndrome and renal agenesis were 14.9% in the early and 10.9% in the later period. Prenatal diagnosis was found in 28.4% of the patients in the early and 34.8% in the later time period and the incidence of intrathoracic liver was 22.4% in the early and 13% in the later time period.

**Table 1:** Patient characteristics in the two different time periods

Patient characteristics	1995–2005 (*n* = 67)	2006–2016 (*n* = 46)	*p* value
Boys	34 (50.8%)	26 (56.5%)	0.570
Girls	33 (49.2%)	20 (43.5%)	
Prenatal diagnosis	19 (28.4%)	16 (34.8%)	0.536
Intrathoracic liver	15 (22.4%)	6 (13%)	0.231
Median gestational age weeks (range)	38 (24–42)	38 (30–42)	0.238
Median birth weight grams (range)	3192 (706–4341)	3215 (1500–4774)	0.147
Major cardiac defects	9 (13.4%)	12 (26.1%)	0.138
Chromosomal abnormalities	2 (3%)	1 (2.2%)	1.000
Other malformations	10 (14.9%)	5 (10.9%)	0.586
Large defect size	26 (40.6%)	14 (31.8%)	0.284
Right sided	13 (19.4%)	6 (13.0%)	0.449
Median age at surgery days (range)	2 (1–3638)	3 (1–1159)	0.937
ECMO	5 (7.5%)	3 (6.5%)	1.000
Mortality rate	12 (17.9%)	2 (4.4%)	0.041

The LHR measurements started in the period between 2006 and 2016. Data on LHR were missing in 6 out of 16 patients with prenatal diagnosis, and were too few to be included in the analysis. At the early time period 40.6% of the patients had a large diaphragmatic defect compared to 31.8% in the later time period (Table 1). Five patients (7.5%) were treated with ECMO in the early and three patients (6.5%) in the later time period. The mortality in those patients was 50%. The mortality rate of 4.4% was significantly lower in the period between 2006 and 2016 compared to 17.9% in the period from 1995 to 2005 as demonstrated in Table 1. During the whole study period from 1995 to 2016, the overall mortality was 12.3% and if neonates who died before surgery were included, the mortality rate was 19.5%. In Table 2, neonates who died prior to surgery or were not candidates for surgical repair are shown. Patient number 1–8 were born during the early time period and number 9 and 10 during the later time period. In patient number 4 and 8, withdrawal of care was done due to trisomy 18.

**Table 2:** Characteristics of neonates who died prior to surgery or were not candidates for surgical repair

Patient	1	2	3	4	5	6	7	8	9	10
Gender	M	M	F	F	M	F	M	F	M	M
Prenatal diagnosis	Yes	Yes	No	No	Yes	Yes	No	No	Yes	Yes
Gestational age (weeks)	37	33	35	40	38	35	37	36	33	25
Birth weight (g)	2350	1950	1790	1981	3420	1460	3000	2200	1770	683
Major cardiac defect	Yes	No	No	Yes	Yes	No	No	Yes	No	Yes
Chromosomal abnormalities	No	No	Yes	Trisomy 18	No	No	No	Trisomy 18	No	No
Other malformations	Fryns syndrome	Multiple^a^	Cornelia de Lange	No	No	Fryns syndrome	No	No	Multiple^b^	No
Side of the defect	Left	Left	Right	Left	Left	Left	Right	Left	Bilat	Left
Age at death (days)	<1	<1	<1	<1	<1	<1	<1	9	<1	<1

^a^Esophageal atresia, myelomeningocele, renal agenesis

^b^Omphalocele, myelomeningocele, bladder exstrophy

**Fig. 1:**
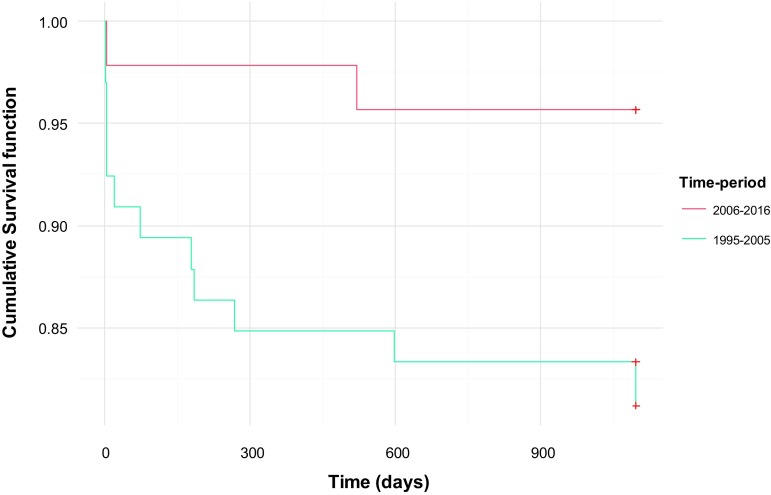
Kaplan–Meier estimates of the cumulative risk of survival up to 900 days in the two different time periods

**Fig. 2:**
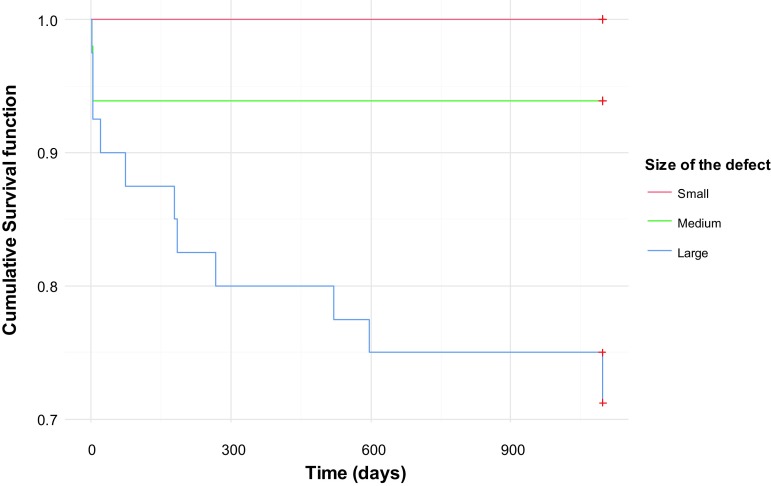
Kaplan–Meier estimates of the cumulative risk of survival up to 900 days in patients with small, medium and large diaphragmatic defects

Patients died earlier after surgery in the early time period (Fig. 1). The survival in patients with a small defect was 100% compared to 72% in patients with a large diaphragmatic defect as demonstrated in Fig. 2. Table 3 demonstrates the potential risk factors included in the univariate Cox proportional hazards model. The risk to die was 6.89 times higher in patients with a large diaphragmatic defect. Patients with an intrathoracic liver had 6.37 times higher risk, infants with a prenatal diagnosis 4.32 and ECMO-treated patients had 6.61 times higher risk to die than patients without those risk factors. Multivariate analysis confirmed a significant association with mortality independent of the other variables for prenatal diagnosis, intrathoracic liver, low Apgar score and low birth weight as demonstrated in Table 4. For every 100 g increase in birth weight, the risk to die was reduced by 7% and for every increase in Apgar score the risk to die was reduced by 34%. Prenatal diagnosis and intrathoracic liver increased the risk to die with 4.55 and 4.0 times, respectively.

**Table 3:** Cox proportional hazards model: univariate analysis

Variable	Coefficient	95% CI	*p* value
Gender	1.57	(0.53–4.70)	0.416
Right-sided CDH	0.93	(0.21–4.17)	0.928
Associated malformations	2.13	(0.74–6.14)	0.161
Age at surgery	0.95	(0.86–1.04)	0.255
Large diaphragmatic defect	6.89	(1.92–24.75)	0.003
Birth weight per 100 g	0.94	(0.88–1.00)	0.034
Gestational age	0.89	(0.78–1.02)	0.088
Prenatal diagnosis	4.32	(1.45–12.91)	0.009
Intrathoracic liver	6.37	(2.00–20.33)	0.002
Apgar 1 min	0.7	(0.55–0.90)	0.004
Apgar 5 min	0.7	(0.55–0.89)	0.004
Apgar 10 min	0.68	(0.52–0.88)	0.003
ECMO	6.61	(2.06–21.13)	0.001

**Table 4:** Multivariate cox proportional hazards model: stepwise based on significant variables in univariate analyses

Variable	Coefficient	95% CI	*p* value
Birth weight per 100 g	0.93	(0.86–1.01)	0.083
Intrathoracic liver	4.00	(1.15–17.20)	0.03
Apgar score	0.66	(0.47–0.86)	0.003
Prenatal diagnosis	4.55	(1.32–19.75)	0.018

## Discussion

Comparing mortality between different reports is confusing as mortality rates are reported for heterogenous populations with CDH from centres with highly varied treatment strategies and volumes of patients. In the study populations, there is a great variation in the proportion of prenatally diagnosed CDH, follow-up time, whether they include only live-born neonates, if they include neonates that die before surgery or only patients that undergo surgery. We have performed this study to be able to compare our results with international reports. During the study period of two decades, there have been changes in both surgical and medical practices. The repair of CDH has changed from a surgical emergency in the beginning of the study period to a planned surgery after stabilization. The first neonate with CDH in our unit that received ECMO was born in the year 2000. High-frequency oscillatory ventilation and inhaled nitric oxide were increasingly used therapies during the study period. Treatments of pulmonary hypertension with sildenafil and bosentan were introduced during the late time period, 2006–2016.

In the present study, prenatal diagnosis, intrathoracic liver, low birth weight and low Apgar score were defined as independent risk factors for mortality. The mortality rate decreased significantly to 4.4% in the later time period even though there was no significant difference in the incidence of the defined risk factors, and it was lower than previously reported [1–3]. Our findings are in accordance with previous reports that found low Apgar score and low birth weight to be strong predictors of mortality [20–22]. The Congenital Diaphragmatic Hernia Study Group (CDHSG) previously published a formula utilizing birth weight and 5-min Apgar score to predict mortality in patients with CDH [20]. However, these factors are not specific for CDH and they have long been associated with adverse outcome in infants with other congenital malformations [23]. Nevertheless, avoidance of prematurity and optimizing preoperative management seem to be important to decrease the mortality in the CDH population.

The intrathoracic liver has been defined as an independent risk factor for mortality [24–28]. It is not surprising as an intrathoracic liver is indicative of a large diaphragmatic defect with early herniation of viscera resulting in severe pulmonary hypoplasia.

The frequency of associated malformations in the current study was higher in the later time period than in a meta-analysis of 102 publications from 1975 to 1998 [10]. In contrast to others, associated malformations were not defined as an independent risk factor in this study [10, 15, 29, 30].

The incidence of prenatal diagnosis was higher in the later time period (35%) and in accordance with the pooled incidence of prenatal diagnosis in a meta-analysis of Skari et al. [10]. It was lower than in an evaluation from 2002 of prenatal diagnosis in 20 European regions with an overall prenatal detection rate of 59% in 187 cases [31]. Prenatal diagnosis was found to be the strongest predictor of mortality in our series of patients as previously reported [10, 32]. The likelihood of prenatal detection increases with large defects with a considerable mediastinal shift and severe pulmonary hypoplasia [10, 32].

The CDHSG reported the defect size to be the strongest predictor of survival [4]. Although a large defect size was associated with higher mortality in the present study it was not found to be an independent risk factor for mortality.

In contrast to a meta-analysis of 2980 patients with CDH, there was no difference in mortality between right- and left-sided CDH in the present study [10].

Permissive hypercapnia and “gentle ventilation” have been reported to increase survival in neonates with CDH [33, 34]. A decrease in the ECMO utilization has been noted in infants managed with permissive hypercapnia and gentle ventilation strategies [33, 34]. A possible explanation for the decreased mortality in the later time period might be that the neonates with CDH were managed in the largest neonatal intensive care unit in the country with a wide experience in “gentle ventilation” in extremely premature neonates. Improved medical treatment of pulmonary hypertension in the later time period might also have contributed to the decreased mortality. Hagadorn et al. found that improved survival in CDH was associated with the increasing use of multiple vasodilators [35].

The benefit of ECMO in the treatment of infants with CDH remains unclear [36]. The efficacy of ECMO in reducing mortality has not been convincing in randomized trials [37, 38]. The incidence of ECMO-treated patients in the present study was low, 7.5 and 6.5% in the early and later time period, respectively, and the mortality rate was 50%. It was the same mortality rate as reported by the ELSO Registry in 2012 [39]. The proportion of neonates requiring ECMO has declined but the mortality rates for neonates supported on ECMO have increased [40, 41].

Our paediatric surgical centre is defined as a low-volume hospital (≤6 cases per year) according to a previous study in 2203 infants with CDH repair [42]. They found higher mortality rates in low-volume hospitals than in high-volume hospitals (>10 cases per year), 23 and 16%, respectively. However, a more recent study in 3738 infants showed no difference in mortality between low- and high-volume hospitals [43].

The strengths of this study are the quite large number of patients and no patients were lost to follow-up. The main weakness of this study is its retrospective design and it is being representative of a single centre.

## Conclusion

In the present study, mortality decreased over time, despite no significant difference in the incidence of independent risk factors and the use of ECMO between the two time periods. The mortality was lower than previously reported. The results indicate that there are many important factors involved to achieve a successful outcome after CDH repair. Large multicentre studies are necessary to define those critical factors and to determine optimal treatment strategies.
